# ERK1/2: A Key Cellular Component for the Formation, Retrieval, Reconsolidation and Persistence of Memory

**DOI:** 10.3389/fnmol.2018.00361

**Published:** 2018-10-05

**Authors:** Jorge H. Medina, Haydee Viola

**Affiliations:** ^1^Instituto de Biología Celular y Neurociencias (IBCN) “Dr Eduardo De Robertis”, CONICET—Universidad de Buenos Aires, Buenos Aires, Argentina; ^2^Departamento de Fisiología, Biología Molecular y Celular “Dr. Hector Maldonado” (FBMC), Facultad de Ciencias Exactas y Naturales, Universidad de Buenos Aires, Buenos Aires, Argentina

**Keywords:** ERK1/2, long-term memory, retrieval, reconsolidation, extinction, durability

## Abstract

Extracellular regulated kinase 1/2 (ERK1/2) has been strongly implicated in several cellular processes. In the brain ERK1/2 activity has been primarily involved in long-term memory (LTM) formation and expression. Here, we review earlier evidence and describe recent developments of ERK1/2 signaling in memory processing focusing the attention on the role of ERK1/2 in memory retrieval and reconsolidation, and in the maintenance of the memory trace including mechanisms involving the protection of labile memories. In addition, relearning requires ERK1/2 activity in selected brain regions. Its involvement in distinct memory stages points at ERK1/2 as a core element in memory processing and as one likely target to treat memory impairments associated with neurological disorders.

## Role of ERK1/2 in Long-Term Memory Formation

A dominant hypothesis emerging in the last 25 years suggests that long-term memory (LTM) formation has two main phases: (1) cellular or synaptic consolidation lasting hours to a couple of days; and (2) systems consolidation which takes days to weeks and comprises the participation of neocortical regions and their interaction with regions of the medial temporal lobe (Squire, 1992; Dudai, 2002). The initial cellular consolidation is thought to involve activation of several neurotransmitter receptors and protein kinase signaling cascades, changes in transcription at the nucleus and translation at the dendritic spines, many posttranslational modifications of synaptic proteins and reorganization of synaptic contacts (McGaugh, 2000; Kandel, 2001; Dudai, 2002; Alberini, 2009).

**Figure 1:**
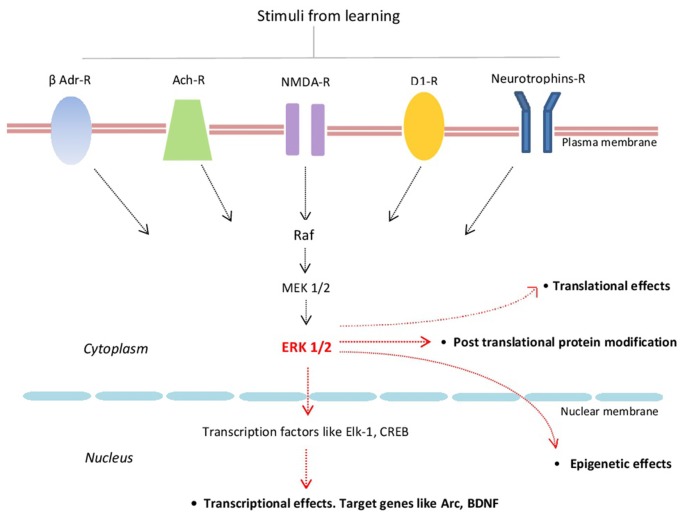
Schematic representation of some of the major components of the extracellular regulated kinase 1/2 (ERK1/2) signaling cascade and its main effects. β-Adr-R, beta adrenergic receptor; Ach-R, muscarinic cholinergic receptors; NMDA-R, NMDA glutamate receptor; D1-R, dopamine 1 receptor; Raf (kinase that phosphorylates MEK1/2 that in turn phosphorylates ERK1/2).

Extracellular regulated kinase 1/2 (ERK1/2; also known as p42/p44 MAPK) are highly conserved protein kinases linking several transmembrane receptors like glutamate NMDA-, cholinergic-, β-adrenergic-, D1 dopamine- and neurotrophin receptors with transcriptional and translational regulation. By activating several transcriptional factors like Elk-1 and CREB, ERKs signaling regulates the expression of several plasticity-related proteins (PRPs) including Arc/Arg3.1 and BDNF (Figure 1; Gutkind, 1998; Sweatt, 2001; Kelleher et al., 2004; Thomas and Huganir, 2004; Bekinschtein et al., 2008; see also Yiannakas and Rosenblum, 2017).

Given that a definite property of LTM is its sensitivity to protein synthesis inhibitors around training (Davis and Squire, 1984; Medina et al., 2008) and that ERK1/2 plays a crucial role in several forms of synaptic plasticity (English and Sweatt, 1997; Rosenblum et al., 2002), it is reasonable to think that ERK1/2 might be involved in many cellular processes including LTM formation (Atkins et al., 1998). After this seminal work demonstrating that contextual fear LTM depends on the activation of ERK1/2 in the dorsal hippocampus, several studies confirmed that this signaling cascade is required in selected brain regions in a variety of learning tasks, including Pavlovian fear conditioning (Schafe et al., 2000), step-down inhibitory avoidance (Walz et al., 1999), learning a novel taste (Swank and Sweatt, 2001), recognition memory (Kelly et al., 2003), spatial Morris water maze (Kelleher et al., 2004), cocaine-induced conditioned place preference (Pan et al., 2011) and conditioned place avoidance (Wang et al., 2017). Most of these studies have also shown that learning was associated with specific changes in the phosphorylation state of ERK1/2. Moreover, some reports showed two waves of ERK1/2 activation after training. One is rapid and transient, peaking about 1–15 min after training and the second one is delayed and persistent lasting for at least 24 h in hippocampus and amygdala (see below “Role of ERK1/2 in Memory Persistence” section; Schafe et al., 2000; Igaz et al., 2004; Trifilieff et al., 2006, 2007; Besnard et al., 2014). It has been reported that the delayed wave of nuclear ERK1/2, CREB and ElK-1 activities are dependent on the activation of NMDA receptor (Figure 1; Cammarota et al., 2000) and that BDNF-induced changes in dendritic spine morphology in hippocampal neurons is mediated by ERK1/2 (Alonso et al., 2004).

The participation of ERK1/2 in synaptic plasticity and memory is not restricted to mammals. Sutton et al. (2001) demonstrated that ERK1/2 participates in an intermediate memory in *Aplysia* whose function seems to be the establishment of LTM, and phosphorylation of ERK1/2 signaling is crucial for LTM formation in the crab (Feld et al., 2005). Also, ERK1/2 homologe is required for memory formation in *Drosophila* (Pagani et al., 2009).

### Could We Dissect the Role of ERK1/2 in Different Processes Leading to Long-Term Memory Formation?

The prevalent view in LTM formation considers the requirement of protein synthesis as a fundamental step of this process (McGaugh, 2000; Kandel, 2001). In that scenario, the activation of ERK1/2 is suggested as part of intracellular signaling cascades involved. However, the recently postulated “behavioral tagging” hypothesis explains the process of LTM formation based on two fundamental and equally important steps: the setting of a transient learning tag and the synthesis of protein (Moncada and Viola, 2007). The idea is based on synaptic tagging and capture hypothesis (Frey and Morris, 1997) which postulated that proteins are used to yield long-lasting changes when they are captured by tags signaling to specific sites activated by the stimuli. Thus, a question arose whether ERK1/2 activation in LTM formation is part of the tag. Under this conceptual framework, a learning experience that induces LTM triggered both processes. However, proteins utilized by tags could also arrive from the synthesis induced by a separated independent event. Therefore, protocols of behavioral tagging take advantage of this fact in order to dissect the setting of the learning tag from the synthesis of PRPs. Original experiments demonstrated that a weak learning only sets its learning tag and could stabilize into LTM by utilizing PRP induced by an associated strong event (for review see Moncada et al., 2015). Because the transient learning tag is triggered by the learning session, the way to study the critical factors involved in its setting and/or maintenance is blocking them around the time of the weak training. The activation of some particular kinases in the hippocampus such as αCAMKII, PKA and ERK1/2 are involved in the formation of LTM from the very first moment after learning, making them interesting candidates as tag components (McGaugh, 2000; Izquierdo et al., 2006). Our results suggest that αCAMKII, PKA and PKMζ play an essential role in the setting of the inhibitory avoidance learning tag, while its machinery does not require the activity of ERKs1/2 neither the synthesis of further proteins (Moncada et al., 2011). In contrast, ERK1/2 kinases have been shown to be required specifically for the setting of synaptic-tags associated with long-term depression (Navakkode et al., 2005; Sajikumar et al., 2007). Thus, the lack of requirement of ERK kinases for the setting of the inhibitory learning-tags is consistent with the idea that avoidance memory might be processed by mechanisms associated with long-term potentiation induction (Whitlock et al., 2006). The activation of ERKs appears to be necessary for providing PRP to induce the formation of LTM (Cammarota et al., 2000). Consistent with these findings is the demonstration that the induction of structural long-term potentiation by activation of few dendritic spines is needed to induce a wave of nuclear ERK activation and therefore gene expression (Zhai et al., 2013). Finally, the intrahippocampal infusion of ERK1/2 inhibitor, but not PKA inhibitor, impaired the effect of novelty exposure on the promotion of contextual fear extinction memory. Thus, hippocampal ERK1/2 may serve as behavioral tags to promote LTM extinction (Liu et al., 2015). Alternatively, the analysis of the results based on “behavioral tagging” hypothesis was recently discussed in terms of non-synaptic mechanisms, like changes in neuronal intrinsic excitability (Korz, 2017).

### Role of ERK1/2 in Short-Term Memory

Much less is known about the role played by ERK1/2 signaling pathway in mammalian short-term memory (STM), mainly because the cellular and molecular mechanisms of STM are not well understood. Typically, STM is referred to as the information store lasting from minutes to few (2–4) hours which is independent of *de novo* protein synthesis and gene expression. The inhibition of ERK1/2 in the dorsal hippocampus immediately after inhibitory avoidance training attenuated STM formation (Alonso et al., 2002; Igaz et al., 2006; see also Giovannini et al., 2015). In addition, blocking hippocampal BDNF function resulted in decreased phosphorylation of ERK2 and impairment of STM while intrahippocampal infusion of recombinant human BDNF increased ERK1/2 activation and facilitated STM (Alonso et al., 2002). On the other hand, in contextual fear STM is insensitive to MEK inhibition (Zamorano et al., 2018).

In *Drosophila*, olfactory conditioning activates ERK1/2 transiently in the mushroom-body neurons. This increased kinase activity occurs 15 min after one trial aversive olfactory learning and significantly prolongs labile STM, mediating active protection of labile memory through maintenance of learning-induced synaptic structural changes (Zhang et al., 2018). The regulation of a labile aversive memory trace by ERK1/2 signaling pathway is bidirectional: its activation sustains the trace for many hours and its inhibition provoked memory decay.

### Role of ERK1/2 on LTM Formation After Spaced Relearning

From the seminal study of Ebbinghaus (1913), we know that the formation of lasting memories benefits from a temporal spaced rest intervals of sessions in contrast to a massed one that involves short or no intervals. This spacing effect has been well demonstrated either in invertebrates and mammals (for a review see Smolen et al., 2016).

Studying in *Aplysia* the tail-elicited siphon withdrawal reflex, Philips et al. (2007) characterized that 45 min, but not of 15 or 60 min, of spacing interval between the electrical shocks applied to the tail, was effective for induction of LTM for sensitization of this defensive reflex. There was also a narrow window of ERK1/2 activation, measured in the tail sensory neurons, 45 min after a single stimulation. If it was disrupted by the exposure to the MEK (kinase that activates ERK1/2) inhibitor U0126, LTM was impaired indicating that this kinase activation is necessary for memory induced by spaced training (Philips et al., 2013). The authors also described that the treatment with serotonin of pleural-pedal ganglia isolated from the mollusk imitated the restricted temporal activation of ERK1/2 observed *in vivo*; this in turn activated p90 RSK kinase and increased the transcription of the immediate early gene ApC/EBP, providing a potential molecular window for memory formation induced by relearning. A computational model based on serotonin-induced PKA and ERK signaling pathways activation, revealed that the efficacy of a protocol to induce long-term facilitation in *Aplysia* is determined by interaction among these kinases activation leading to CREB1 phosphorylation (Zhang et al., 2011).

Working on another invertebrate model, Pagani et al. (2009) trained *Drosophila* flies with a standard olfactory conditioning consisting of pairing odor with an electric shock. They demonstrated that the cycle of ERK1/2 activation is involved in defining the duration of resting intervals necessary for LTM induction. In that sense, ERK1/2 activation must decay enough to permit a resetting with the subsequent trial during spaced training, being the tyrosine phosphatase SHP2 a key factor involved. The spacing effect is a phenomenon which also affects structural plasticity. In this sense, analyzing the number of new synaptic boutons at the single-synapse level after distinct patterns of stimulation in motoneurons of *Drosophila*, it was showed that suppressing or enhancing ERK1/2 signaling changed how synapses decode a pattern of stimuli (San Martin et al., 2017).

Another kind of memory that responds to the spacing effect is fear potentiated startle memory. Training rats with two-trial light-shock pairing with inter trials ranged between 45 min and 3 days resulted in the expression of a robust LTM (Parsons and Davis, 2012). Amygdala ERK1/2 activation 1 h after training was suggested as part of the mechanism of metaplasticity that permits the formation of persistent memory. The role of ERK1/2 activation in relearning was also demonstrated in inhibitory avoidance. This hippocampus-dependent aversive paradigm showed additional learning induced by a second training session performed 1 day after the first one. However, this additional memory does not involve the hippocampus but, instead, the striatum. The infusion of a MEK inhibitor into striatum, both at the time of second training and 3 h later, caused the impairment of this memory enhancement (Cammarota et al., 2005). Synaptic ERK1/2 activation was also associated with formation of object location memory in fragile X syndrome model in mice (Seese et al., 2014). In this case, three short-spaced trials separated by 1 h elevated pERK1/2 in the septotemporal segment of the hippocampus; and the inactivation of ERK1/2 before the last trial blocked LTM.

## Role of ERK1/2 in Memory Retrieval

Retrieval is the only way to measure memory (James, 1890). Although considerable efforts have been made in elucidating the molecular signatures associated with the acquisition of new information, much less is known about the molecular signaling events that accompany memory retrieval. This general assertion does not fit well when one considers the role of ERK1/2 activity on the expression of memory. There are plenty of studies showing the importance of this signaling cascade in memory retrieval and its consequences, extinction and reconsolidation (see later Role of MAPK in reconsolidation). Inhibition of ERK1/2 activation before testing 24 or 48 h after training abolished expression of aversive (Szapiro et al., 2000; Chen et al., 2005; Sindreu et al., 2007; Besnard et al., 2013, 2014; Zamorano et al., 2018) and spatial memories (Zhang et al., 2004). Expression of aversive memory is hindered by pretest administration of MEK inhibitor into the prelimbic (Luo et al., 2017) and anterior cingulate cortex (Barros et al., 2000).

Retrieval-induced ERK1/2 activation has been also observed in several species and in different learning tasks. For instance, ERK activity increases after inhibitory avoidance retention test in rats, and this increase is proportional to the amount of retrieval (Szapiro et al., 2000). Using immunohistochemical analysis, Besnard et al. (2014) demonstrated that after retrieval of contextual fear conditioning (0–30 min) there were clear-cut increases in the activation of ERK1/2 signaling in the dentate gyrus and CA1, but not in CA3, region of the hippocampus. Also they observed selected changes in ERK1/2 activity in some nuclei of the amygdala. Based probably in the differences in the timing of postrecall animals sacrifice, those findings are in partial agreement with those recently published by Zamorano et al. (2018): recall of contextual fear conditioning is accompanied by a selective increase in the phosphorylation state of ERK1/2 in CA1 pyramidal neurons, but not in CA3 and dentate gyrus neurons. Those hyperphosphorylated neurons of the CA1 regions exhibited also ERK1/2 activation during training, suggesting that CA1 ERK1/2 participates in encoding contextual information of emotional value.

The role of ERK1/2 signaling is not restricted to spatial or fear memories involving the hippocampal formation and related cortical regions. Pretest infusion of MEK inhibitor into the nucleus accumbens abolished retrieval of cocaine- conditioned place preference and prevented the recall-induced increase in the phosphorylation state of ERKs, CREB, Elk-1 and the expression of c-fos (Miller and Marshall, 2005).

## Role of ERK1/2 in Memory Reconsolidation and Extinction

Two main consequences of memory retrieval are reconsolidation and extinction. These are two different and mutually exclusive processes resulting from the labilization of the memory trace by unreinforced retrieval and both require ERK1/2 activation (see below). There is also a transitional state of the original memory after reactivation, baptized as limbo, with no evidence of reconsolidation or extinction in which ERK1/2 signaling plays no role (Merlo et al., 2014, 2018).

Reconsolidation refers to as the process of LTM destabilization/stabilization after retrieval involving posttranslational changes and gene expression regulation. The participation of ERK1/2 in memory reconsolidation was first demonstrated by Kelly et al. (2003) who found that ERK1/2 signaling in the hippocampus is important to reconsolidate recognition memory. After that, using different aversive as well as appetitive learning tasks others confirmed that ERK1/2 signaling cascade in selected brain regions is required for reconsolidation (Duvarci et al., 2005; Miller and Marshall, 2005; Krawczyk et al., 2015). Combining pharmacological and genetic approaches, Cestari et al. (2006) found that ERK2 played a pivotal role in reconsolidation of cued fear conditioning. The last three above-mentioned works obtained results consistent with a role of ERK1/2 in memory restabilization, while two more recent works (Besnard et al., 2014; Zamorano et al., 2018) suggested that in addition to restabilization, ERK1/2 participates in memory destabilization. It is important to note that these mechanisms vary across brain regions and learning tasks. One explanation for this discrepancy may involve activation of different pools of ERK1/2. A good example consistent with this idea is the work of Merlo et al. (2018) who suggest that NMDA receptor-dependent activation of different pools of amygdalar ERK1/2 may be required for reconsolidation and extinction of fear conditioning.

Extinction is the learned inhibition of the expression of previously acquired memories (Izquierdo et al., 2016). In other words, retrieval performed in the absence of an unconditioned stimulus gives rise to a fading process called extinction (Pavlov, 1956). Fear extinction revealed by a decrease in fear after non-reinforced trials require ERK1/2 activation and is associated with specific modifications in the phosphorylation state of hippocampal ERK1/2 (Szapiro et al., 2003; Fischer et al., 2007). Similar findings were obtained in amygdala-dependent learning tasks where the increase in ERK1/2 activation is NMDA receptor-dependent (Merlo et al., 2014, 2018; see Cestari et al., 2014 for references). In addition, a novel ERK-S6K1-GluA1 signaling cascade in amygdala is critically involved in extinction (Huynh et al., 2018). A third brain region is crucial for extinction memory formation: the medial prefrontal cortex. ERK1/2 signaling in this region appears to modulate extinction consolidation and retrieval (see Izquierdo et al., 2016).

## Role of ERK1/2 in Memory Persistence

Besides the role of retrieval consequences in memory persistence (see “Role of ERK1/2 in Memory Reconsolidation and Extinction” section), there are other mechanisms involving ERK1/2 that modulate (see also “Role of ERK1/2 in Short-Term Memory” section) memory durability. In 2008, two studies showed that ERK/MAPK signaling is crucial for the maintenance of long lasting memory storage in rodents. One of the works demonstrated that the activation of ERK1/2 by the neurotrophin BDNF is important for the persistence of two aversive learning tasks (Bekinschtein et al., 2008). This signaling cascade activation occurs around 12 h after training and is consistent with a previous finding showing an increase in the phosphorylation state of ERK2 late after inhibitory avoidance training (Igaz et al., 2004). What are the downstream targets of ERK1/2 for maintaining the memory trace? Two immediate early genes, c-fos and egr-1, exhibited an increased expression between 12 h and 24 h after training that is blocked by inhibiting the BDNF/ERK1/2 signaling pathway (Bekinschtein et al., 2007; Katche et al., 2010, 2012). The other study demonstrated a circadian oscillation of the phosphorylation state of ERK1/2 and CREB in the hippocampus after contextual fear conditioning (Eckel-Mahan et al., 2008). The authors found that disruption of this oscillation by inhibition of ERK1/2 at the peak of ERK1/2 activity blocks its oscillation and hinders the persistence of contextual fear memory. In addition, disruption of ERKs oscillation by exposing the mice to constant light conditions also impairs memory persistence. These findings suggest that memory persistence may depend on the lasting oscillation of ERK1/2/CREB transcriptional signaling pathways during the circadian cycle (Eckel-Mahan, 2012).

It has been indicated that memory reconsolidation is a required step for the strengthening of hippocampus-dependent contextual fear memory, thus supporting a role of ERK1/2 activation in memory durability (Lee, 2008). Moreover, inhibition of ERK1/2 signaling 3 h after memory reactivation did not affect memory reconsolidation when tested 24 h after retrieval, but greatly impaired performance when tested 7 days apart (Krawczyk et al., 2016). Also, pretest blockade of ERK1/2 signaling abolished fear memory expression and relearning-induced strengthening (Roesler and Quevedo, 2009). In addition, in the fly Zhang et al. (2018) suggest that a conserved mechanism involving ERK signaling is important for memory persistence (see also “Role of ERK1/2 in Short-Term Memory” section).

## Conclusion

Neurons possess different transmembrane neurotransmitter receptors which respond to synaptic activity and are coupled to the activation of ERK1/2 kinases. As a consequence, changes occur in gene transcription, protein translation and posttranslational modification that are required in information processing. Brain makes an internal representation of our experiences and it encodes, stores, and retrieves information in a dynamic way. Here, we summarized the role of ERK1/2 activation across the memory life, focusing on recent advances in the field of memory formation and relearning, extinction, reconsolidation and persistence of memory storage. From this compilation of data, it emerges a main conclusion: ERK1/2 activity in selected brain regions is a core and evolutionary conserved cellular element of memory processing. Future advances in this field include the study of downstream targets of ERKs activation (receptors, ion channels and factors affecting protein synthesis, trafficking and degradation), as well as the evaluation of novel inhibitors of ERK1/2 pathway in the prevention or treatment of neurological and psychiatric diseases involving memory disorders.

## Author Contributions

JM and HV designed and wrote the manuscript.

## Conflict of Interest Statement

The authors declare that the research was conducted in the absence of any commercial or financial relationships that could be construed as a potential conflict of interest.

## References

[B1] Alberini C. M. (2009). Transcription factors in long-term memory and synaptic plasticity. Physiol. Rev. 89, 121–145. 10.1152/physrev.00017.2008 19126756 PMC3883056

[B3] Alonso M. Medina J. H. Pozzo-Miller L. (2004). ERK1/2 activation is necessary for BDNF to increase dendritic spine density in hippocampal CA1 pyramidal neurons. Learn. Mem. 11, 172–178. 10.1101/lm.67804 15054132 PMC379687

[B2] Alonso M. Vianna M. R. Depino A. M. Mello e Souza T. Szapiro G. Izquierdo I.. (2002). BDNF-triggered events in the rat hippocampus are required for both short- and long-term memory formation. Hippocampus 12, 551–560. 10.1002/hipo.10035 12201640

[B4] Atkins C. M. Selcher J. C. Petraitis J. J. Trzaskos J. M. Sweatt J. D. (1998). The MAPK cascade is required for mammalian associative learning. Nat. Neurosci. 1, 602–609. 10.1038/2836 10196568

[B5] Barros D. M. Izquierdo L. A. Mello e Souza T. Ardenghi P. G. Pereira P. Medina J. H.. (2000). Molecular signalling pathways in the cerebral cortex are required for retrieval of one-trial avoidance learning in rats. Behav. Brain Res. 114, 183–192. 10.1016/s0166-4328(00)00226-6 10996059

[B6] Bekinschtein P. Cammarota M. Igaz L. M. Bevilaqua L. R. Izquierdo I. Medina J. H. (2007). Persistence of long-term memory storage requires a late protein synthesis- and BDNF-dependent phase in the hippocampus. Neuron 53, 261–277. 10.1016/j.neuron.2006.11.025 17224407

[B7] Bekinschtein P. Cammarota M. Katche C. Slipczuk L. Rossato J. I. Goldin A.. (2008). BDNF is essential to promote persistence of long-term memory storage. Proc. Natl. Acad. Sci. U S A 105, 2711–2716. 10.1073/pnas.0711863105 18263738 PMC2268201

[B8] Besnard A. Caboche J. Laroche S. (2013). Recall and reconsolidation of contextual fear memory: differential control by ERK and Zif268 expression dosage. PLoS One 8:e72006. 10.1371/journal.pone.0072006 23977192 PMC3745394

[B9] Besnard A. Laroche S. Caboche J. (2014). Comparative dynamics of MAPK/ERK signalling components and immediate early genes in the hippocampus and amygdala following contextual fear conditioning and retrieval. Brain Struct. Funct. 219, 415–430. 10.1007/s00429-013-0505-y 23389809

[B10] Cammarota M. Bevilaqua L. R. Ardenghi P. Paratcha G. Levi de Stein M. Izquierdo I.. (2000). Learning-associated activation of nuclear MAPK, CREB and Elk-1, along with Fos production, in the rat hippocampus after a one-trial avoidance learning: abolition by NMDA receptor blockade. Mol. Brain Res. 76, 36–46. 10.1016/s0169-328x(99)00329-0 10719213

[B11] Cammarota M. Bevilaqua L. R. Köhler C. Medina J. H. Izquierdo I. (2005). Learning twice is different from learning once and from learning more. Neuroscience 132, 273–279. 10.1016/j.neuroscience.2005.01.022 15802182

[B12] Cestari V. Costanzi M. Castellano C. Rossi-Arnaud C. (2006). A role for ERK2 in reconsolidation of fear memories in mice. Neurobiol. Learn. Mem. 86, 133–143. 10.1016/j.nlm.2006.01.003 16504549

[B13] Cestari V. Rossi-Arnaud C. Saraulli D. Costanzi M. (2014). The MAPK of fear: from memory consolidation to memory extinction. Brain Res. Bull. 105, 8–16. 10.1016/j.brainresbull.2013.09.007 24080449

[B14] Chen X. Garelick M. G. Wang H. Lil V. Athos J. Storm D. R. (2005). PI3 kinase signaling is required for retrieval and extinction of contextual memory. Nat. Neurosci. 8, 925–931. 10.1038/nn1482 15937483

[B15] Davis H. P. Squire L. R. (1984). Protein synthesis and memory: a review. Psychol. Bull. 96, 518–559. 10.1037//0033-2909.96.3.518 6096908

[B16] Dudai Y. (2002). Molecular bases of long-term memories: a question of persistence. Curr. Opin. Neurobiol. 12, 211–216. 10.1016/s0959-4388(02)00305-7 12015239

[B17] Duvarci S. Nader K. LeDoux J. E. (2005). Activation of extracellular signal-regulated kinase mitogen-activated protein kinase cascade in the amygdala is required for memory reconsolidation of auditory fear conditioning. Eur. J. Neurosci. 21, 283–289. 10.1111/j.1460-9568.2004.03824.x 15654867

[B18] Ebbinghaus H. (1913). Memory: A Contribution to Experimental Psychology, Chapter 2 Teachers Colleges. New York, NY: Columbia University.

[B19] Eckel-Mahan K. L. (2012). Circadian oscillations within the hippocampus support memory formation and persistence. Front. Mol. Neurosci. 5:46. 10.3389/fnmol.2012.00046 22529773 PMC3328119

[B20] Eckel-Mahan K. L. Phan T. Han S. Wang H. Chan G. C. Scheiner Z. S.. (2008). Circadian oscillation of hippocampal MAPK activity and cAmp: implications for memory persistence. Nat. Neurosci. 11, 1074–1082. 10.1038/nn.2174 19160506 PMC2772165

[B21] English J. D. Sweatt J. D. (1997). A requirement for the mitogen-activated protein kinase cascade in hippocampal long term potentiation. J. Biol. Chem. 272, 19103–19106. 10.1074/jbc.272.31.19103 9235897

[B22] Feld M. Dimant B. Delorenzi A. Coso O. Romano A. (2005). Phosphorylation of extra-nuclear ERK/MAPK is required for long-term memory consolidation in the crab Chasmagnathus. Behav. Brain Res. 158, 251–261. 10.1016/j.bbr.2004.09.005 15698891

[B23] Fischer A. Radulovic M. Schrick C. Sananbenesi F. Godovac-Zimmermann J. Radulovic J. (2007). Hippocampal Mek/Erk signaling mediates extinction of contextual freezing behavior. Neurobiol. Learn. Mem. 87, 149–158. 10.1016/j.nlm.2006.08.003 16979915 PMC1839930

[B24] Frey U. Morris R. G. M. (1997). Synaptic tagging and long-term potentiation. Nature 385, 533–536. 10.1038/385533a0 9020359

[B25] Giovannini M. G. Lana D. Pepeu G. (2015). The integrated role of ACh, ERK and mTOR in the mechanisms of hippocampal inhibitory avoidance memory. Neurobiol. Learn. Mem. 119, 18–33. 10.1016/j.nlm.2014.12.014 25595880

[B26] Gutkind J. S. (1998). The pathways connecting G protein-coupled receptors to the nucleus through divergent mitogen-activated protein kinase cascades. J. Biol. Chem. 273, 1839–1842. 10.1074/jbc.273.4.1839 9442012

[B27] Huynh T. N. Santini E. Mojica E. Fink A. E. Hall B. S. Fetcho R. N.. (2018). Activation of a novel p70 S6 kinase 1-dependent intracellular cascade in the basolateral nucleus of the amygdala is required for the acquisition of extinction memory. Mol. Psychiatry 23, 1394–1401. 10.1038/mp.2017.99 28461701 PMC5668214

[B28] Igaz L. M. Bekinschtein P. Izquierdo I. Medina J. H. (2004). One-trial aversive learning induces late changes in hippocampal CaMKIIα, Homer 1a, Syntaxin 1a and ERK2 protein levels. Mol. Brain Res. 132, 1–12. 10.1016/j.molbrainres.2004.08.016 15548423

[B29] Igaz L. M. Winograd M. Cammarota M. Alonso M. Izquierdo I. Medina J. H. (2006). Early activation of extracellular signal-regulated kinase signaling pathway in the hippocampus is required for short-term memory formation of a fear-motivated learning. Cell. Mol. Neurobiol. 26, 989–1002. 10.1007/s10571-006-9116-y 16977492 PMC11520636

[B30] Izquierdo I. Bevilaqua L. R. M. Rossato J. Bonini J. S. Medina J. H. Cammarota M. (2006). Different molecular cascades in different sites of the brain control memory consolidation. Trends Neurosci. 29, 496–505. 10.1016/j.tins.2006.07.005 16872686

[B31] Izquierdo I. Furini C. R. Myskiw J. C. (2016). Fear memory. Physiol. Rev. 96, 695–750. 10.1152/physrev.00018.2015 26983799

[B32] James W. (1890). Principles of Psychology. New York, NY: Henry Holt & Company.

[B33] Kandel E. R. (2001). The molecular biology of memory storage: a dialogue between genes and synapses. Science 294, 1030–1038. 10.1126/science.1067020 11691980

[B34] Katche C. Bekinschtein P. Slipczuk L. Goldin A. Izquierdo I. Cammarota M.. (2010). Delayed wave of c-Fos expression in the dorsal hippocampus involved specifically in persistence of long-term memory storage. Proc. Natl. Acad. Sci. U S A 107, 349–354. 10.1073/pnas.0912931107 20018662 PMC2806699

[B35] Katche C. Goldin A. Gonzalez C. Bekinschtein P. Medina J. H. (2012). Maintenance of long-term memory storage is dependent on late posttraining Egr-1 expression. Neurobiol. Learn. Mem. 98, 220–227. 10.1016/j.nlm.2012.08.001 22906840

[B36] Kelleher R. J. Govindarajan A. Jung H. Y. Kang H. Tonegawa S. (2004). Translational control by MAPK signaling in long-term synaptic plasticity and memory. Cell 116, 467–479. 10.1016/s0092-8674(04)00115-1 15016380

[B37] Kelly A. Laroche S. Davis S. (2003). Activation of mitogen-activated protein kinase/extracellular signal-regulated kinase in hippocampal circuitry is required for consolidation and reconsolidation of recognition memory. J. Neurosci. 23, 5354–5360. 10.1523/JNEUROSCI.23-12-05354.2003 12832561 PMC6741214

[B100] Korz V. (2017). Behavioral tagging: Synaptic event or cellular alteration? Neurobiol. Learn. Mem. 148, 8–10. 10.1016/j.nlm.2017.12.005 29277582

[B38] Krawczyk M. C. Blake M. G. Baratti C. M. Romano A. Boccia M. M. Feld M. (2015). Memory reconsolidation of an inhibitory avoidance task in mice involves cytosolic ERK2 bidirectional modulation. Neuroscience 294, 227–237. 10.1016/j.neuroscience.2015.03.019 25791227

[B39] Krawczyk M. C. Navarro N. Blake M. G. Romano A. Feld M. Boccia M. M. (2016). Reconsolidation-induced memory persistence: participation of late phase hippocampal ERK activation. Neurobiol. Learn. Mem. 133, 79–88. 10.1016/j.nlm.2016.06.013 27321160

[B40] Lee J. L. (2008). Memory reconsolidation mediates the strengthening of memory by additional learning. Nat. Neurosci. 11, 1264–1266. 10.1038/nn.2205 18849987

[B41] Liu J. F. Yang C. Deng J. H. Yan W. Wang H. M. Luo Y. X.. (2015). Role of hippocampal β-adrenergic and glucocorticoid receptors in the novelty-induced enhancement of fear extinction. J. Neurosci. 35, 8308–8321. 10.1523/JNEUROSCI.0005-15.2015 26019344 PMC6605349

[B42] Luo F. Zheng J. Sun X. Deng W. K. Li B. M. Liu F. (2017). Prelimbic cortex extracellular signal-regulated kinase 1/2 activation is required for memory retrieval of long-term inhibitory avoidance. Brain Res. 1661, 88–99. 10.1016/j.brainres.2017.02.010 28214522

[B43] McGaugh J. L. (2000). Memory—a century of consolidation. Science 287, 248–251. 10.1126/science.287.5451.248 10634773

[B44] Medina J. H. Bekinschtein P. Cammarota M. Izquierdo I. (2008). Do memories consolidate to persist or they persist to consolidate? Behav. Brain Res. 192, 61–69. 10.1016/j.bbr.2008.02.006 18374993

[B46] Merlo E. Milton A. M. Everitt B. J. (2018). A novel retrieval-dependent memory process revealed by the arrest of ERK1/2 activation in the basolateral amygdala. J. Neurosci. 28, 3199–3207. 10.1523/JNEUROSCI.3273-17.2018 29476015 PMC6596053

[B45] Merlo E. Milton A. L. Goozée Z. Y. Theobald D. E. Everitt B. J. (2014). Reconsolidation and extinction are dissociable and mutually exclusive processes: behavioral and molecular evidence. J. Neurosci. 34, 2422–2431. 10.1523/JNEUROSCI.4001-13.2014 24523532 PMC3921417

[B47] Miller C. A. Marshall J. F. (2005). Molecular substrates for retrieval and reconsolidation of cocaine-associated contextual memory. Neuron 47, 873–884. 10.1016/j.neuron.2005.08.006 16157281

[B48] Moncada D. Ballarini F. Martinez M. C. Frey J. U. Viola H. (2011). Identification of transmitter systems and learning tag molecules involved in behavioral tagging during memory formation. Proc. Natl. Acad. Sci. U S A 108, 12931–12936. 10.1073/pnas.1104495108 21768371 PMC3150922

[B49] Moncada D. Ballarini F. Viola H. (2015). Behavioral tagging: a translation of the synaptic tagging and capture hypothesis. Neural Plast. 2015:650780. 10.1155/2015/650780 26380117 PMC4562088

[B50] Moncada D. Viola H. (2007). Induction of long-term memory by exposure to novelty requires protein synthesis: evidence for a behavioral tagging. J. Neurosci. 27, 7476–7481. 10.1523/JNEUROSCI.1083-07.2007 17626208 PMC6672624

[B52] Navakkode S. Sajikumar S. Frey J. U. (2005). Mitogen-activated protein kinase-mediated reinforcement of hippocampal early long-termdepression by the type IV-specific phosphodiesterase inhibitor rolipram and its effect on synaptic tagging. J. Neurosci. 25, 10664–10670. 10.1523/JNEUROSCI.2443-05.2005 16291939 PMC6725844

[B53] Pagani M. R. Oishi K. Gelb B. D. Zhong Y. (2009). The phosphatase SHP2 regulates the spacing effect for long-term memory induction. Cell 139, 186–198. 10.1016/j.cell.2009.08.033 19804763 PMC2770243

[B54] Pan B. Zhong P. Sun D. Liu Q. S. (2011). Extracellular signal-regulated kinase signaling in the ventral tegmental area mediates cocaine-induced synaptic plasticity and rewarding effects. J. Neurosci. 31, 11244–11255. 10.1523/JNEUROSCI.1040-11.2011 21813685 PMC3153072

[B55] Parsons R. G. Davis M. A. (2012). A metaplasticity-like mechanism supports the selection of fear memories: role of protein kinase a in the amygdala. J. Neurosci. 32, 7843–7851. 10.1523/JNEUROSCI.0939-12.2012 22674260 PMC3375025

[B56] Pavlov I. P. (1956). Conditioned Reflexes. New York, NY: Dover Corporation.

[B57] Philips G. T. Tzvetkova E. I. Carew T. J. (2007). Transient mitogen-activated protein kinase activation is confined to a narrow temporal window required for the induction of two-trial long-term memory in *Aplysia*. J. Neurosci. 27, 13701–13705. 10.1523/JNEUROSCI.4262-07.2007 18077681 PMC6673619

[B58] Philips G. T. Ye X. Kopec A. M. Carew T. J. (2013). MAPK establishes a molecular context that defines effective training patterns for long-term memory formation. J. Neurosci. 33, 7565–7573. 10.1523/JNEUROSCI.5561-12.2013 23616561 PMC3865502

[B59] Roesler R. Quevedo J. (2009). Retrieval mediated by hippocampal extracellular signal-regulated kinase/mitogen-activated protein kinase is required for memory strengthening. Neuroscience 160, 711–715. 10.1016/j.neuroscience.2009.03.024 19298845

[B60] Rosenblum K. Futter M. Voss K. Erent M. Skehel P. A. French P.. (2002). The role of extracellular regulated kinases I/II in late-phase long-term potentiation. J. Neurosci. 22, 5432–5441. 10.1523/JNEUROSCI.22-13-05432.2002 12097495 PMC6758197

[B61] Sajikumar S. Navakkode S. Frey J. U. (2007). Identification of compartment- and process-specific molecules required for ‘synaptic tagging’ during long-term potentiation and long-term depression in hippocampal CA1. J. Neurosci. 27, 5068–5080. 10.1523/JNEUROSCI.4940-06.2007 17494693 PMC6672381

[B62] San Martin A. Rela L. Gelb B. Pagani M. R. (2017). The spacing effect for structural synaptic plasticity provides specificity and precision in plastic changes. J. Neurosci. 37, 4992–5007. 10.1523/JNEUROSCI.2607-16.2017 28432141 PMC5426186

[B63] Schafe G. E. Atkins C. M. Swank M. W. Bauer E. P. Sweatt J. D. LeDoux J. E. (2000). Activation of ERK/MAP kinase in the amygdala is required for memory consolidation of pavlovian fear conditioning. J. Neurosci. 20, 8177–8187. 10.1523/JNEUROSCI.20-21-08177.2000 11050141 PMC6772720

[B64] Seese R. R. Wang K. Yao Y. Q. Lynch G. Gall C. M. (2014). Spaced training rescues memory and ERK1/2 signaling in fragile X syndrome model mice. Proc. Natl. Acad. Sci. U S A 111, 16907–16912. 10.1073/pnas.1413335111 25385607 PMC4250145

[B65] Sindreu C. B. Scheiner Z. S. Storm D. R. (2007). Ca^2+^-stimulated adenylyl cyclases regulate ERK-dependent activation of MSK1 during fear conditioning. Neuron 53, 79–89. 10.1016/j.neuron.2006.11.024 17196532 PMC1858648

[B66] Smolen P. Zhang Y. Byrne J. H. (2016). The right time to learn: mechanisms and optimization of spaced learning. Nat. Rev. Neurosci. 17, 77–88. 10.1038/nrn.2015.18 26806627 PMC5126970

[B67] Squire L. R. (1992). Memory and the hippocampus: a synthesis from findings with rats, monkeys, and humans. Psychol. Rev. 99, 195–231. 10.1037//0033-295x.99.2.195 1594723

[B68] Sutton M. A. Masters S. E. Bagnall M. W. Carew T. J. (2001). Molecular mechanisms underlying a unique intermediate phase of memory in *Aplysia*. Neuron 31, 143–154. 10.1016/s0896-6273(01)00342-7 11498057

[B69] Swank M. W. Sweatt J. D. (2001). Increased histone acetyltransferase and lysine acetyltransferase activity and biphasic activation of the ERK/RSK cascade in insular cortex during novel taste learning. J. Neurosci. 21, 3383–3391. 10.1523/JNEUROSCI.21-10-03383.2001 11331368 PMC6762472

[B70] Sweatt J. D. (2001). The neuronal MAP kinase cascade: a biochemical signal integration system subserving synaptic plasticity and memory. J. Neurochem. 76, 1–10. 10.1046/j.1471-4159.2001.00054.x 11145972

[B71] Szapiro G. Izquierdo L. A. Alonso M. Barros D. Paratcha G. Ardenghi P.. (2000). Participation of hippocampal metabotropic glutamate receptors, protein kinase A and mitogen-activated protein kinases in memory retrieval. Neuroscience 99, 1–5. 10.1016/s0306-4522(00)00236-0 10924946

[B72] Szapiro G. Vianna M. R. McGaugh J. L. Medina J. H. Izquierdo I. (2003). The role of NMDA glutamate receptors, PKA, MAPK, and CAMKII in the hippocampus in extinction of conditioned fear. Hippocampus 13, 53–58. 10.1002/hipo.10043 12625457

[B73] Thomas G. M. Huganir R. L. (2004). MAPK cascade signalling and synaptic plasticity. Nat. Rev. Neurosci. 5, 173–183. 10.1038/nrn1346 14976517

[B74] Trifilieff P. Calandreau L. Herry C. Mons N. Micheau J. (2007). Biphasic ERK1/2 activation in both the hippocampus and amygdala may reveal a system consolidation of contextual fear memory. Neurobiol. Learn. Mem. 88, 424–434. 10.1016/j.nlm.2007.05.004 17613254

[B75] Trifilieff P. Herry C. Vanhoutte P. Caboche J. Desmedt A. Riedel G.. (2006). Foreground contextual fear memory consolidation requires two independent phases of hippocampal ERK/CREB activation. Learn. Mem. 13, 349–358. 10.1101/lm.80206 16705140 PMC1475817

[B76] Walz R. Roesler R. Barros D. M. de Souza M. M. Rodrigues C. Sant’Anna M. K.. (1999). Effects of post-training infusions of a mitogen-activated protein kinase kinase inhibitor into the hippocampus or entorhinal cortex on short- and long-term retention of inhibitory avoidance. Behav. Pharmacol. 10, 723–730. 10.1097/00008877-199912000-00003 10780287

[B77] Wang X. Zhang L. Zhan Y. Li D. Zhang Y. Wang G.. (2017). Contribution of BDNF/TrkB signalling in the rACC to the development of pain-related aversion via activation of ERK in rats with spared nerve injury. Brain Res. 1671, 111–120. 10.1016/j.brainres.2017.07.010 28732667

[B78] Whitlock J. R. Heynen A. J. Shuler M. G. Bear M. F. (2006). Learning induces long-term potentiation in the hippocampus. Science 313, 1093–1097. 10.1126/science.1128134 16931756

[B79] Yiannakas A. Rosenblum K. (2017). The insula and taste learning. Front. Mol. Neurosci. 10:335. 10.3389/fnmol.2017.00335 29163022 PMC5676397

[B80] Zamorano C. Fernández J. Storm D. Carné X. Sindreu C. (2018). Memory retrieval re-activates Erk1/2 signaling in the same set of CA1 neurons recruited during conditioning. Neuroscience 370, 101–111. 10.1016/j.neuroscience.2017.03.034 28366664

[B81] Zhai S. Ark E. Parra-Bueno P. Yasuda R. (2013). Long-distance integration of nuclear ERK signaling triggered by activation of a few dendritic spines. Science 342, 1107–1111. 10.1126/science.1245622 24288335 PMC4318497

[B82] Zhang Y. Liu R. Y. Heberton G. A. Smolen P. Baxter D. A. Cleary L. J.. (2011). Computational design of enhanced learning protocols. Nat. Neurosci. 15, 294–297. 10.1038/nn.2990 22197829 PMC3267874

[B84] Zhang X. Li Q. Wang L. Liu Z. Zhong Y. (2018). Active protection: learning-activated Raf/MAPK activity protects labile memory from Rac1-independent forgetting. Neuron 98, 142.e4–155.e4. 10.1016/j.neuron.2018.02.025 29551489

[B83] Zhang H. T. Zhao Y. Huang Y. Dorairaj N. R. Chandler L. J. O’Donnell J. M. (2004). Inhibition of the phosphodiesterase 4 (PDE4) enzyme reverses memory deficits produced by infusion of the MEK inhibitor U0126 into the CA1 subregion of the rat hippocampus. Neuropsychopharmacology 29, 1432–1439. 10.1038/sj.npp.1300440 15114341

